# MnS Incorporation into Higher Manganese Silicide Yields a Green Thermoelectric Composite with High Performance/Price Ratio

**DOI:** 10.1002/advs.201800626

**Published:** 2018-07-03

**Authors:** Zhiliang Li, Jin‐Feng Dong, Fu‐Hua Sun, Asfandiyar, Yu Pan, Shu‐Fang Wang, Qing Wang, Dan Zhang, Lei Zhao, Jing‐Feng Li

**Affiliations:** ^1^ Hebei Key Lab of Optic‐Electronic Information and Materials College of Physics Science and Technology Hebei University Baoding 071002 P. R. China; ^2^ State Key Laboratory of New Ceramics and Fine Processing School of Materials Science and Engineering Tsinghua University Beijing 100084 P. R. China

**Keywords:** higher manganese silicide, MnS, performance/price ratios, thermoelectric materials

## Abstract

Thermoelectric materials that can directly convert heat to electrical energy offer a viable solution for reducing the usage of fossil energy by harvesting waste heat resources. Higher manganese silicide (HMS) is a naturally abundant, eco‐friendly, and low‐cost p‐type thermoelectric semiconductor with high power factor (PF); however, its figure of merit (*ZT*) is limited by intrinsically high thermal conductivity (κ). For effectively enhancing the thermoelectric performance of HMS and avoiding the use of expensive or toxic elements, such as Re, Te, or Pb, a green p‐type MnS with high Seebeck coefficient (*S*) and low κ is incorporated into the HMS matrix to form MnS/HMS composites. The incorporation of MnS leads to a 31% reduction of κ and a 10% increase of *S*. The *ZT* value increases by ≈48% from 0.40 to 0.59 at 823 K. Correspondingly, performance/price ratio is first proposed to evaluate the practical value of thermoelectric materials, which is higher than those of the vast majority of current thermoelectric materials. This study provides an overview of enhancing *ZT* of HMS and reducing costs, which may also be applicable to other thermoelectric materials.

Thermoelectric (TE) materials have attracted considerable attention owing to their capabilities of directly converting heat to electrical energy.^1^, ^2^, ^3^, ^4^ In general, TE performance can be estimated via the dimensionless figure of merit *ZT* = *S* ^2^ *σT*/κ_tot_, where *S*, σ, *T*, and κ_tot_ are the Seebeck coefficient, electrical conductivity, absolute temperature, and total thermal conductivity, respectively.^5^, ^6^, ^7^, ^8^ At present, PbTe‐based compounds^9^, ^10^ and SnSe single crystals^11^, ^12^ are identified as two typically efficient TE materials at medium temperature, with *ZT*_max_ reaching high values of 2.2 and 2.6, respectively. However, several intrinsic tasks, such as the utilization of the toxic Pb element, complicate preparation processes, and poor thermal or chemical stabilities are expected to be difficult to overcome. By contrast, among all mid‐temperature TE materials, higher manganese silicide (HMS) is known as a naturally abundant, eco‐friendly, and low‐cost TE semiconductor with satisfactory thermal stability and good mechanical strength. The optimal power factor (PF =*S* ^2^σ) of pure HMS is about 1.5 × 10^−3^ W m^−1^ K^−2^; this value is higher than that of SnSe single crystal,^11^, ^12^ In‐doped Cu_2_Se (*ZT* = 2.6)^13^ and even comparable to those of PbTe_0.7_S_0.3_ 14 or Ge_0.87_Pb_0.13_Te^15^ alloys, the *ZT* values of which exceed 2.0 after certain modifications.

However, state‐of‐the‐art research^16^, ^17^, ^18^ has revealed the *ZT*_max_ values of pure HMS to be only about 0.2–0.4 due to its intrinsically high total thermal conductivity (κ_tot_, ≈3.0 ± 0.2 W m^−1^ K^−1^). At present, significant efforts have been focused on decreasing the κ_tot_ of HMS. Shi and co‐workers^19^, ^20^ demonstrated that κ_tot_ can be decreased to 2.2 W m^−1^ K^−1^ at 773 K when certain 50–200 nm ReSi_1.75_ precipitates formed in the HMS samples; consequently, *ZT* increased from 0.45 to 0.57. Takeuchi and co‐workers^21^ proved that κ_tot_ can be further decreased to 1.8 W m^−1^ K^−1^ at 773 K when 6.0 at% Re was used to form a supersaturated Re solid solution HMS, and the corresponding *ZT* value increased to 0.9 at 773 K. In our previous work,^22^ Te nanowires were selected to prepare MnTe/HMS nano/bulk structures, resulting in 38% reduction of κ_tot_ and ≈71% increase in *ZT*. In addition, other strategies have been applied to effectively decrease κ_tot_ of HMS, including element doping to increase lattice defects,^23^, ^24^, ^25^, ^26^, ^27^, ^28^ intensifying grain refining to increase grain boundaries,^29^, ^30^, ^31^, ^32^ and precipitating nanoinclusions to intensify phonon scattering^19^, ^33^, ^34^, ^35^, ^36^, ^37^, ^38^, ^39^; these strategies resulted in enhanced *ZT* from 0.40 to 0.65. However, some limitations remain with modification and future applications. First, certain elements that can theoretically improve TE performance, including Te or S, cannot replace Si atoms because of the relatively stable lattice structure. Second, electrical transport properties are adversely affected a lot when reduce κ_tot_ via element doping or precipitating inclusions. Third, most effective strategies that are currently available use rare, toxic, and expensive elements, such as Re, Te, Cr, and Ru, which result in a relatively high manufacturing costs and serious environmental pollution. Thus, in this study, we attempted to modify HMS by using the green and cheap compound MnS. Both light S‐doped and MnS‐combined effects were achieved when a small amount of MnS was incorporated into HMS; thus, the final κ_tot_ decreased by ≈31% with a slight effect on electrical transport properties. Correspondingly, performance/price ratio was first estimated and demonstrated to be higher than those of most state‐of‐the‐art TE materials.

Pure HMS (Mn_15_Si_26_, **Figure** **1**A, line a) was prepared using a facile, short‐time, and low‐energy route of wet ball milling combined with spark plasma sintering (SPS).^22^ Formation of impure phase‐semimetallic MnSi and monatomic Si was prevented in all MnS/HMS composite specimens. As a reference, pure MnS bulk (Figure 1A, line e) was obtained using the same sintering condition. None of MnS peaks was observed, indicating no MnS crystals were left when 0.5 at% MnS was added to HMS (Figure 1A, line b). Probably, a high consumption of MnS happened due to the S‐doped effect, resulting in the majority of S atoms embeds into the HMS crystal cells. However, the two main specific diffractive peaks of MnS (200) and (220) planes gradually increased and was intensified with the increase in MnS concentration to 1.0 at% (Figure 1A, line c) and 2.0 at% (Figure 1A, line d); this finding indicated that certain MnS inclusions were precipitated at these proportions. Lattice parameters *a* and *c* of the (HMS)_1−_ *_x_*(MnS)*_x_* composites were calculated from the carefully refined high precision step‐scan X‐ray diffraction (XRD) patterns using a generalized analytical software (Jade 6.5) according to the following equations

(1) $$n\lambda \;=\;2d_{hkl}\sin\theta$$

(2) $$\frac{1}{d_{hkl}{}^{2}}=\frac{h^{2}+k^{2}}{a^{2}}\;+\frac{l^{2}}{c^{2}}$$

**Figure 1:**
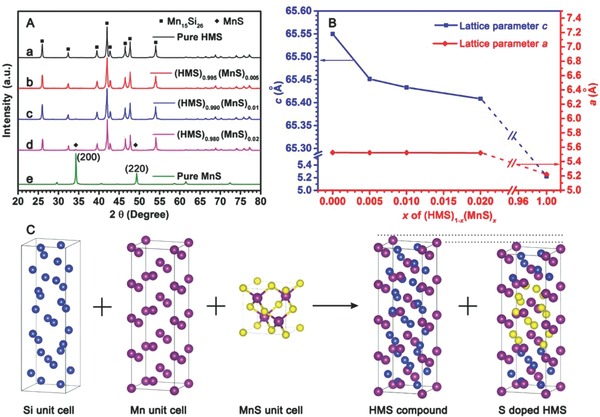
A) XRD patterns and B) lattice parameters of (HMS)_1−_ *_x_*(MnS)*_x_* with different proportions of MnS. C) simulative formation mechanism of HMS and S‐doped HMS.

Where *n* = 1, λ is the wavelength of the diffraction wave (1.5406 Å), *h*, *k*, and *l* are the indices of the crystallographic plane (the highest diffraction peak indexes (21*15*)), *d_hkl_* is the interplanar spacing. For HMS tetragonal phase, α  =  β = γ  = 90°, *a* = *b* ≠ *c*. The peaks shifts (i.e., angular deflections (2θ)) of the three highest diffraction peaks lead to the variation of *d_hkl_* and hence the parameters *a* and *c*. When MnS amount increased from 0 to 2.0 at%, no obvious fluctuation occurred on the lattice parameter *a* (Figure 1B, red line) because of similar values between HMS (*a* = 5.525 Å) and MnS (*a* = 5.220 Å). However, the lattice parameter *c* changed a lot due to the intrinsically big differences between pure HMS (*c* = 65.550 Å) and MnS (*c* = 5.220 Å). Parameter *c* rapidly decreased from 65.550 to 65.452 Å (2θ of the highest diffraction peak increases ≈0.03°) with the MnS increasing from 0 to 0.5 at%, wherein the decreasing rate (defines as Δ*c*/Δat%, where at% is the atomic percentage of MnS/HMS) is ≈19.6 Å per pct. (pct. is the abbreviation of percentage). The sharp decrease of parameter *c* indicates that several Si sites in HMS were possibly substituted by smaller S atoms. This is also the reason why no MnS peaks were observed when MnS concentration was lower than 0.5 at%. And then, the parameter *c* decreased slowly from 65.452 to 65.408 Å (2θ of the highest diffraction peak increases ≈0.02˚) with MnS increasing from 0.5 to 2.0 at% (Figure 1B, blue line), which corresponds to a decreasing rate of only ≈2.93 Å per pct. Different from the traditional strategy that uses elementary substance as raw material, S‐doped effect was observed in this case. Corresponding mechanisms can be described using Figure 1C and the following chemical formulas

(3) $$\mathrm{Mn}+1.733\mathrm{Si}\to {\mathrm{MnSi}}_{1.733}\left({\mathrm{Mn}}_{15}{\mathrm{Si}}_{26},\;\mathrm{HMS}\right)$$

(4) $$0.995\mathrm{Mn}+1.725\mathrm{Si}+0.005\mathrm{MnS}\to {\mathrm{MnSi}}_{1.725}S_{0.005}$$

The chemical valences of Mn and S elements are tentatively unsaturated in the MnS compound. Thus, both elements can continue reacting with extra Si and Mn elementary substances, respectively. Meanwhile, Mn–S chemical bond was retained in the final crystal lattice due to its intrinsic high binding energy. Several small‐sized S atoms (smaller than Si atoms) were embedded into HMS lattice and decreased the lattice parameter *c*. However, some MnS crystals precipitated with further addition of MnS raw materials due to limited S accommodation in HMS specimens prepared using short‐time sintering technique.

The overall morphologies of (HMS)_1−_ *_x_*(MnS)*_x_* with different proportions of MnS are shown in the corresponding scanning electron microscopy (SEM) images (**Figure** **2**A–D). Similar to pure HMS (Figure 2A), 0.5 at% MnS‐added HMS showed no impurity phase (Figure 2B), which possibly indicated that the doping effect is the dominant mechanism in this case. Some white nanocrystals were precipitated when 1.0 at% MnS was added (Figure 2C). The grain sizes of precipitates increased to micrometer scale with the addition of 2.0 at% MnS. The (HMS)_0.98_(MnS)_0.02_ sample was analyzed using energy dispersive spectrometry (EDS) to accurately confirm the ingredients of the white precipitates (Figure 2E–H). Mn was uniformly distributed, whereas certain S enriched regions and corresponding Si enriched areas were observed in Figure 2F,G, indicating that the white precipitates were residual MnS crystals. By contrast, S elements not only existed in white precipitates but were also uniformly dispersed in the HMS matrix (Figure 2F), thereby implying that certain Si sites in HMS were homogeneously substituted by S atoms. Further detailed structural information was provided in the magnified transmission electron microscopy (TEM) (Figure 2I–L). Contrastive EDS between Points I and II was conducted and is shown in Figure 2I,L. At Point I, the EDS peak of the S element was too weak to be observed due to its low accommodation in the HMS matrix (Figure 2J). However, an inverse phenomenon occurred at Point II: the EDS peak of S considerably increased with decreasing Si element (Figure 2K), indicating that the inclusions are mainly composed of MnS. Interestingly, a relatively compatible structure without massive lattice distortion was observed when the grain sizes of the MnS inclusions were lower than 100 nm (Figure 2I,M). Inversely, certain grain boundaries that started from the corner of MnS crystal were formed due to relatively high deformational strain (Figure 2L,N). Furthermore, the pores increased in quantity and size with increasing MnS, as shown in the SEM images (Figure 2A–D), because of its possible pinning effect in the S‐doped HMS sample or incompatibility between MnS and HMS in (HMS)_1−_ *_x_*(MnS)*_x_* composites. The corresponding porosity was also proved by the relative densities (**Table** **1**), which decreased from 95.25% to 94.72% with the *x* increasing from 0 to 0.02. Some nanopores, which were supposedly effective on medium‐frequency phonon scattering, were also recorded in the final TEM images (Figure 2I).

**Figure 2:**
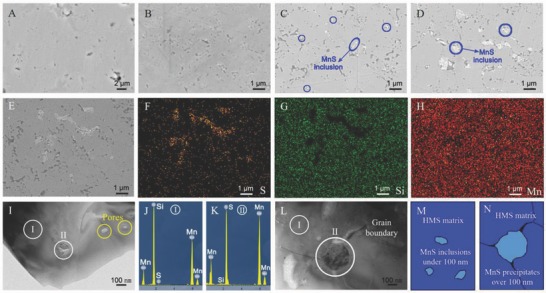
A–D) SEM images of (HMS)_1−_ *_x_*(MnS)*_x_* composites with different nominal proportions of MnS, with *x* ranging from 0 to 2.0 at%. E–H) overall morphology of the (HMS)_0.98_(MnS)_0.02_ sample and the corresponding EDS spectra on F) S, G) Si, and H) Mn. I–L) TEM images, EDS spectra, and simulated structure of (HMS)_1−_ *_x_*(MnS)*_x_* composites.

**Table 1:** Theoretical, experimental, and relative densities of (HMS)_1−_ *_x_*(MnS)*_x_* composites

	Theoretical density [g cm^−3^]	Experimental density [g cm^−3^]	Relative density [%]
Pure HMS	5.158	4.913	95.25
(HMS)_0.995_(MnS)_0.005_	5.152	4.889	94.90
(HMS)_0.990_(MnS)_0.010_	5.146	4.876	94.75
(HMS)_0.980_(MnS)_0.020_	5.134	4.863	94.72

For determining the effect of MnS, the TE coefficients of (HMS)_1−_ *_x_*(MnS)*_x_* composites were tested. The Seebeck coefficient (*S*) at 823 K rapidly increased from 206 to 236 µV K^−1^ when MnS gradually increased from 0 to 2.0 at% (**Figure** **3**A). Carrier concentration (*p*) were calculated using *p* = 1/(*eR*_H_), where *R*_H_ is the Hall coefficient measured via a professional measurement system based on the Hall effect. The *p* decreased considerably when a small amount of MnS (0.5 at%) was added (Figure 3B, line a). However, the decrease rate declined when a relatively high proportion of MnS (1.0 or 2.0 at%) was used. Thus, different enhancing mechanisms occurred even though *S* is directly proportional to the MnS percentage. In the system with low initial MnS concentration, several Si atoms in HMS were possibly substituted by S, and a few electrons were released as shown in Equation (5). By contrast, the unsaturated Mn^2+^ ions belonging to MnS compounds continued to react with Si to form new Mn–Si chemical bonds, which lead to the transformation of Mn^2+^ ions into Mn^7+^ and loss of electrons (Equation (6)). As a result, Mn–S and Mn–Si chemical bonds coexisted for Mn elements.

(5) $$S^{2-}\overset{{\mathrm{Mn}}_{\mathrm{15}}{\mathrm{Si}}_{\mathrm{26}}}{\to }S_{\mathrm{Si}}\prime \prime +2e^{-}$$

(6) $${\mathrm{Mn}}^{\mathrm{2+}}\to {\mathrm{Mn}}^{\mathrm{7+}}+{\mathrm{5e}}^{-}$$

**Figure 3:**
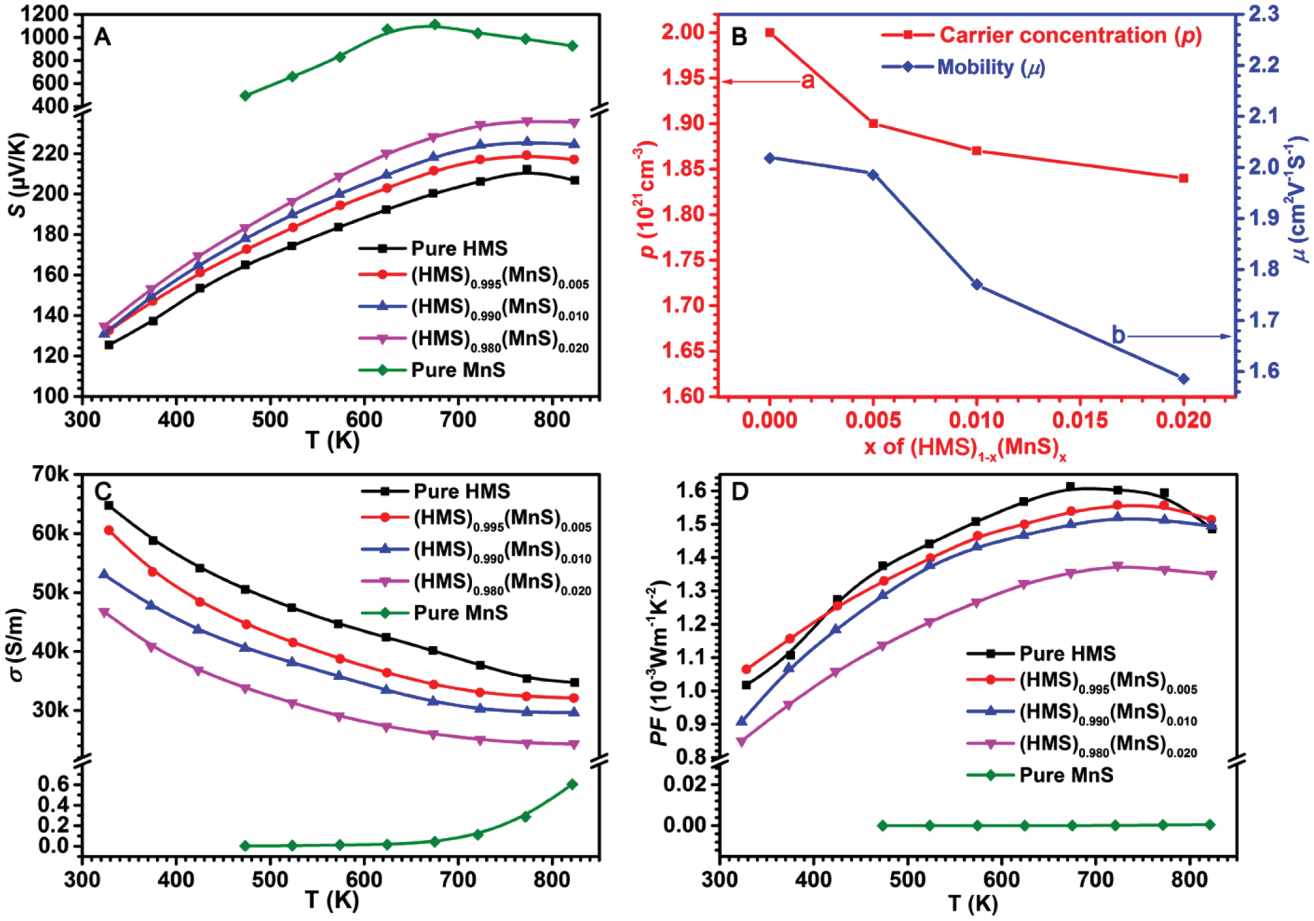
A) Seebeck coefficient, C) electrical conductivity, and D) *PF* of (HMS)_1−_ *_x_*(MnS)*_x_* composites as a function of temperature. B) carrier concentration and mobility of (HMS)_1−_ *_x_*(MnS)*_x_* composites with *x* ranging from 0 to 2.0 at%.

Several holes in the bulk were neutralized by the released electrons, resulting in rapidly reduced carrier concentration in the bulk. Given that SPS is a low‐energy, ultrashort‐time sintering technique, S cannot totally replace Si atoms when a high proportion of MnS (1.0 or 2.0 at%) was added. The redundant MnS crystals remained in the final bulks. In this case, carrier concentration decreased gradually (Figure 3B, line a). However, an opposite moderating trend in carrier mobility was observed (Figure 3B, line b). When the addition ratio of MnS was under 0.5 at%, the element doping effect was the dominant mechanism in the sample. The corresponding lattice structure (Figure 1A, line b) or microtopography (Figure 2B) did not change a lot comparing with the pure HMS (Figure 1A, line a and Figure 2A), resulting in ≈2.0% decrease of mobility. When MnS increased to 1.0 or 2.0 at%, the amount of point defect, grain boundary, and inclusion increased rapidly, and the corresponding mobility considerably decreased due to the gradually deteriorated carrier channels resulted from the amount and size increase of pores and inclusions (Figure 2A–D). Moreover, given the ultrahigh *S* of MnS (Figure 3A, green line), *S* increases with further addition of MnS. However, electrical conductivity (σ) is an arithmetic product (σ = *neµ*) of carrier concentration (*n*) and mobility (*µ*). Simultaneous reduction in *n* and *µ* leads to decreased σ (Figure 3C). The poor electrical transport property of MnS (Figure 3C, green line) exerts a certain negative effect on the final electrical conductivity and results in gradual decrease in σ of ≈31% from 35 to 24 kSm^−1^ at 823 K with the addition of 2.0 at% MnS. Ultimately, by considering the increasing *S* and decreasing σ, the corresponding power factor (PF = *S* ^2^σ, Figure 3D) slightly decreased when a small amount of MnS was added (0.5 or 1.0 at%). Even so, these values (1.48–1.52 mW m^−1^ K^−2^ at 823 K) are comparable to those reported in state‐of‐the‐art publications (1.31–1.79 mW m^−1^ K^−2^ at 823 K).^17^, ^19^, ^30^, ^33^ However, PF considerably decreased due to rapidly decreased σ when MnS continuously increased to 2.0 at% (Figure 3D, pink line).

Moreover, the total thermal conductivities (κ_tot_) were significantly affected when a certain amount of MnS was added (**Figure** **4**A). Compared with that of pure HMS, κ_tot_ of the (HMS)_0.995_(MnS)_0.005_ composite decreased by about 20% from 3.03 to 2.42 W m^−1^ K^−1^ at 823 K (Figure 4A, black and red lines). Violent scatterings possibly occurred on phonons with short wavelength because of the rapidly increased dislocations and point defects,^40^, ^41^ which resulted from lattice mismatch between MnS and HMS. κ_tot_ continued to decrease to 2.1 and 2.0 W m^−1^ K^−1^ at 823 K when additional MnS was used (1.0 and 2.0 at%, Figure 4A, blue and pink lines, respectively). Interestingly, the marked decrease that generally appears after 675 K weakened in a stepwise manner with increasing MnS amount. In theory, the second phase with relatively high energy gap (*E*_g,MnS_, ≈3.7 eV) may decrease the bipolar effect of matrix (*E*_gHMS_, ≈0.77 eV) and correspondingly reduce thermal conductivity at high temperature. Similar phenomena have been experimentally proved by some state‐of‐the‐art studies in PbSe and ZnTe based composite.^42^, ^43^ As a reference, the κ_tot_ of pure MnS bulk was displayed in Figure 4A, green line. The substantial decrease in κ_tot_ of the MnS compound also contributed to the decrease in final κ_tot_ of the (HMS)_0.995_(MnS)_0.005_ composite at high temperature. κ_tot_ mainly consists of lattice thermal conductivity (κ_L_) and electrical thermal conductivity (κ_e_), which is respectively contributed from the conduction of phonons and charge carriers. For most semiconductors, κ_L_ is comparable to κ_e_ and cannot be neglected as in metals.^5^ The κ_L_ is obtained from κ_L_ = κ − κ_e_, where κ_e_ can be calculated by the universally applicable Wiedemann–Franz law, κ_e_ = *LσT*, in which *L* is the Lorenz number.^44^, ^45^, ^46^, ^47^, ^48^ For HMS with slight change in composition (assuming a single parabolic band and acoustic phonon scattering domination),^47^ *L* is calculated to be ≈1.54 × 10^−8^ V^2^ K^−2^,^19^, ^49^ which is lower than the Sommerfeld value of 2.45 × 10^−8^ V^2^ K^−2^ because of the intrinsically metallic limit (lower Fermi level, as manifested by the increasing Seebeck coefficient)^19^ as well as the very inelastic carrier scattering.^50^ The corresponding κ_L_ and κ_e_ are described in Figure 4B (solid lines and dotted lines, respectively). The patterns show that the largest contribution to the final κ_tot_ originated from κ_L_ because of the relatively high value and similar variation trend in κ_tot_. For evaluating how low κ_L_ is, a theoretical κ_L min_ of pure HMS (Figure 4B, yellow line with pentagrams) was provided using the following equation proposed by Cahill et al.^51^

(7) $$\kappa_{L\;\min}={\left(\frac{\pi }{6}\right)}^{1/3}\;\kappa_{B}n^{2/3}\sum_{i}\nu_{i}{\left(\frac{T}{\theta_{i}}\right)}^{2}\int_{0}^{\theta_{i}/T}\frac{x^{3}e^{x}}{{\left(e^{x}-1\right)}^{2}}dx$$

where κ_B_, *n*, ν_i_, and θ_i_ are the Boltzmann constant, number density of atoms, speed of sound,^19^ and cutoff frequency for each polarization, respectively. As shown by the solid curves, κ_L_ at high temperature exceeding 625 K gradually converges to κ_L min_ of HMS at MnS addition exceeding 1.0 at%. For specimens with high MnS concentration, in addition to dislocations and point defects, extra MnS nanoinclusions, nanopores, and grain boundaries resulting from high strain of the large MnS precipitates (Figure 4E) contributed to the scattering of phonons with middle or long wavelength. Ultimately, with the slightly decreased PF and rapidly reduced κ_tot_ taken into consideration, the optimal *ZT* value of (HMS)_1−_ *_x_*(MnS)*_x_* composites finally increased by ≈48% from 0.40 (pure HMS) to 0.59 at 823 K (Figure 4C) when 1.0 at% MnS was used; these findings are comparable to those of state‐of‐the‐art studies on HMS‐based materials.^16^, ^17^, ^18^, ^19^, ^23^, ^24^, ^25^, ^26^, ^29^, ^30^, ^31^, ^35^, ^36^, ^37^, ^38^ In addition to performance, the prices of raw materials of the TE composites should not be neglected for the future applications. The components of (HMS)_1−_ *_x_*(MnS)*_x_* composite are all low‐cost elements; thus, corresponding prices should be considered in the final criteria of evaluation. Here, a judgment equation named performance/price ratio (ξ) for TE materials was first proposed. The ξ of the *A_a_B_b_*…*N_n_* compound can be calculated using the following formula

(8) $$\begin{array}{c} \xi \;\text{=}\frac{\text{TE}\;\text{performance}}{\text{Unit}\;\text{price}\;\text{of}\;\text{compound}}\\ \text{=}\frac{ZT}{\sum \frac{xM_{X}}{M_{A_{a}B_{b}\ldots N_{n}}}\times P_{m,X}}\;\\ \text{=}\frac{ZT}{\frac{aM_{A}}{M_{A_{a}B_{b}\ldots N_{n}}}\times P_{m,\;A}+\frac{bM_{B}}{M_{A_{a}B_{b}\ldots N_{n}}}\times P_{m,\;B}+\ldots +\frac{nM_{N}}{M_{A_{a}B_{b}\ldots N_{n}}}\times P_{m,\;N}} \end{array}$$

where *X* is a certain element of the *A_a_B_b_*…*N_n_* compound, *M* is the mole mass of the selected element of the *A_a_B_b_*…*N_n_* compound, and *P_m_* is the element price based on unit mass. The most optimal *ZT* values and corresponding ξ of typical TE materials from low to high temperature are listed in Figure 4D.^8^, ^10^, ^11^, ^21^, ^52^, ^53^, ^54^, ^55^ Although *ZT* of the (HMS)_0.99_(MnS)_0.01_ composite was the lowest among the selected TE representatives, its corresponding ξ is higher than those of the most other materials. Moreover, by considering manufacturing cost (short‐time wet ball milling combined with energy‐saved spark plasma sintering), ξ is expected to further increase. In addition, reliable mechanical strength and good thermal or chemical stability may render the (HMS)_1−_ *_x_*(MnS)*_x_* composites as a potentially practical TE material.

**Figure 4:**
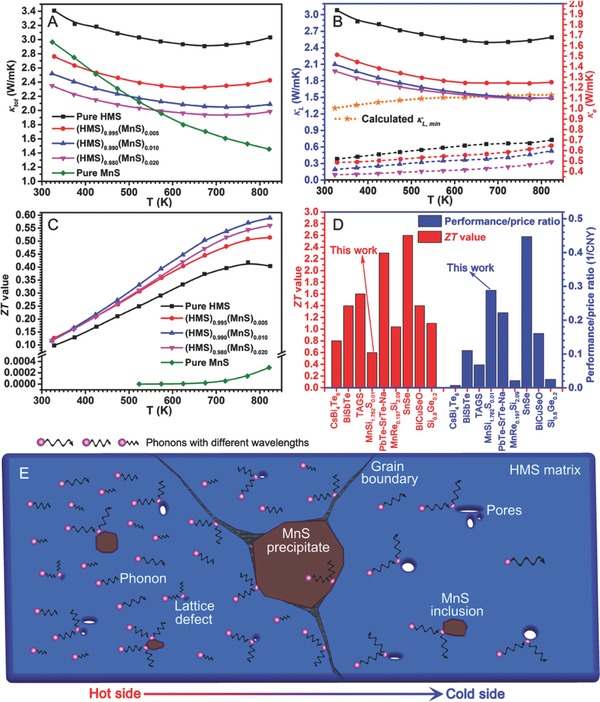
A) Total thermal conductivities, B) lattice thermal conductivity, and electrical thermal conductivity. C) *ZT* values of (HMS)_1−_ *_x_*(MnS)*_x_* composites. D) The most optimal *ZT* values and corresponding performance/price ratio from low to high temperature. E) Possible phonon scattering mechanism in the (HMS)_1−_ *_x_*(MnS)*_x_* composite.

In this study, significant enhancement in *ZT* value (≈48% from 0.40 to 0.59 at 823 K) was achieved when 1.0 at% MnS was used for incorporation into HMS. Performance/price ratio, an evaluation criterion, was first proposed for TE materials, and the corresponding value of (HMS)_0.99_(MnS)_0.01_ composite was higher than those of most TE materials at present. The total thermal conductivities of (HMS)_1−_ *_x_*(MnS)*_x_* composites were rapidly decreased by enhanced phonon scattering effect resulting from increased dislocations, point defects, extra MnS nanoinclusions, nanopores, and grain boundaries. PF decreased slightly when a small amount of MnS was added into HMS. The low‐cost elementary composition and energy‐saving preparation technologies offer a new option for future potential applications in TE devices.

## Experimental Section

Proportional manganese (Mn, 99.9%), silicon (Si, 99.99%), manganous sulfide (MnS, 99.99%) powders, and 40 mL of *n*‐hexane were homogeneously mixed via planetary ball milling for 2 h at a rotational speed of 350 rpm in a zirconia jar. Subsequently, the muddy mixture was adequately dried to powder in a vacuum oven at 60 °C for 4 h and then sintered to 1050 °C for 6 min in SPS (SPS‐211Lx, Fuji Electronic Industrial Co., Ltd, Japan) under an axial pressure of 60 MPa and a vacuum of 3.0 Pa. Disks of 1.5 mm thickness and rectangular columns with a size of 7 × 2.5 × 2.5 mm were sliced along the direction perpendicular and parallel to the axial pressure for evaluating thermal conductivities and electrical transport properties, respectively.

The crystal phases and morphologies of the as‐prepared specimens were characterized using a Bruker AXS XRD‐D8 Focus X‐ray diffractometer and field emission scanning electron microscopy (Zeiss Merlin). High‐resolution transmission electron microscopy (HRTEM) images were recorded via a JEOL JEM 2010F field emission transmission electron microscope. Seebeck coefficient and resistivity were measured using a ZEM‐3 Seebeck coefficient/electric resistance measuring system (Ulvac‐Riko, Inc.). The total thermal conductivities were calculated using the equation κ_tot_ = *λC*_p_ *d*, where λ, *C*_p_, and *d* are thermal diffusion coefficient, specific heat capacity, and density, respectively. Thermal diffusion coefficient was evaluated using a laser flash apparatus (TC‐9000, Ulvac‐Riko). Specific heat capacities were measured using a DSC STA449 equipment in reference to the value calculated using the Dulong–Petit law. Hall carrier concentration (*p*) and mobility (*µ*) were measured using a Hall measurement system (ResiTest 8340DC, Tokyo).

## Conflict of Interest

The authors declare no conflict of interest.
